# Preparation in the business and practice of medicine: perspectives from recent gynecologic oncology graduates and program directors

**DOI:** 10.1186/s40661-017-0051-z

**Published:** 2017-09-22

**Authors:** Matthew Schlumbrecht, John Siemon, Guillermo Morales, Marilyn Huang, Brian Slomovitz

**Affiliations:** 1 Division of Gynecologic Oncology, Sylvester Comprehensive Cancer Center, University of Miami, 1121 NW 14th St, Suite 345C, Miami, FL 33136 USA; 2 Department of Obstetrics and Gynecology, University of Miami, Miami, USA

**Keywords:** Fellowship education, Program directors, Survey, Gynecologic oncology

## Abstract

**Background:**

Preparation in the business of medicine is reported to be poor across a number of specialties. No data exist about such preparation in gynecologic oncology training programs. Our objectives were to evaluate current time dedicated to these initiatives, report recent graduate perceptions about personal preparedness, and assess areas where improvements in training can occur.

**Methods:**

Two separate surveys were created and distributed, one to 183 Society of Gynecologic Oncology candidate members and the other to 48 gynecologic oncology fellowship program directors. Candidate member surveys included questions about perceived preparedness for independent research, teaching, job-hunting, insurance, and billing. Program director surveys assessed current and desired time dedicated to the topics asked concurrently on the candidate survey. Statistical analysis was performed using Chi-squared (or Fisher’s exact test if appropriate) and logistic regression.

**Results:**

Survey response rates of candidate members and program directors were 28% and 40%, respectively. Candidate members wanted increased training in all measures except retrospective protocol writing. Female candidates wanted more training on writing letters of intent (LOI) (*p* = 0.01) and billing (*p* < 0.01). Compared to their current schedules, program directors desired more time to teach how to write an investigator initiated trial (p = 0.01). 94% of program directors reported having career goal discussions with their fellows, while only 72% of candidate members reported that this occurred (*p* = 0.05).

**Conclusion:**

Recent graduates want more preparation in the non-clinical aspects of their careers. Reconciling program director and fellow desires and increasing communication between the two may serve to achieve the educational goals of each.

**Electronic supplementary material:**

The online version of this article (10.1186/s40661-017-0051-z) contains supplementary material, which is available to authorized users.

## Background

Gynecologic oncology training programs have evolved much over the last ten years, and the volume of information that fellows are required to master has significantly increased. As it is has been reported that matriculating fellows are often ill-equipped for the rigors of a gynecologic oncology fellowship [1], there are increasing pressures on training programs to ensure adequate surgical experiences while providing thorough didactic teaching in a short amount of time. Education in the non-clinical aspects of medicine, including skills to be a successful academician, experience with trial development, and exposure to different types of practice environments, may be lacking in lieu of basic science and clinical programs. At a time when recent fellowship graduates report dissatisfaction with their fellowship didactic lectures [2], it is important to understand the areas where they perceive weaknesses, and address these concerns in order to work towards further optimization of training programs. Our objective was to evaluate the perceptions of recent fellowship graduates in regards to their preparation for the non-clinical responsibilities of medicine, to evaluate current fellowship program education for such non-clinical responsibilities, and to determine if fellowship program directors are themselves satisfied with their own content curricula.

## Methods

Approval was obtained from the University of Miami Institutional Review Board. Two separate de novo surveys were created (Additional files 1 and 2). The first survey was for recent gynecologic oncology fellowship graduates who had not yet passed the oral certification exam in gynecologic oncology. These individuals are candidates for membership in the Society of Gynecologic Oncology (SGO), and were identified by a query of the SGO member database. The candidate survey contained questions about demographics, education history, and current practice setting. Additional questions about perceived preparation for a number of issues in post-training practice were posed in yes/no format, and addressed comfort with grant writing, protocol writing, effective teaching, billing and coding, malpractice insurance and medical-legal issues, financial planning, and disability; follow-up questions about these topics asked whether or not they would have preferred more training on each. Candidate members were also asked about how they obtained their first jobs, including faculty mentorship during the process, use of a headhunter, and contract review.

The second survey was developed for current gynecologic oncology fellowship program directors. As the subspecialty of gynecologic oncology is currently undergoing a transition in oversight from the American Board of Obstetrics and Gynecology (ABOG) to the Accreditation Council for Graduate Medical Education (ACGME), program directors were identified by a query of both organizations. Associate program directors were not included. Length of the program was ascertained. The survey then inquired about current number of didactic hours dedicated to each of the following topics over the length of fellowship training: protocol writing, drafting a grant proposal/letter of intent, being an effective teacher, billing/coding, medical-legal concerns, financial planning, disability, and the Affordable Care Act. Program directors were then asked how many hours they would *prefer* to spend on each of these topics.

The surveys for both program directors and candidate members were released via email in January, 2017, with a link to the questionnaire. The survey link was active for six weeks, and three separate email invitations to participate were sent. A statement of implied informed consent was included in the survey instructions. Responses were collected anonymously and stored in a RedCap database.

Statistical analyses were completed using STATA IC (StataCorp, College Station, TX). All data was used, even if the surveys were not completed in toto. Summary statistics were generated to describe the cohort. Chi-square testing (or Fisher’s exact when appropriate) was used to analyze proportional associations between groups. Logistic regression was used to estimate associations between binary variables. All tests were two-sided, and *p*-values <0.05 were considered statistically significant.

## Results

Of the 183 candidate members contacted, fifty-one (28%) completed the survey. Demographic characteristics are shown in Table 1. Seventy one percent (71%) of the respondents were female, and the median age was 36 years (range 32–44). The majority had completed their fellowships after 2013, with 75% attending three-year programs. Nearly 57% of participants were in university-based practices, while 33% reported being employed in community/academic hybrid programs, and 10% in community based programs.

**Table 1:** Demographics of candidate member respondents (*n* = 51)

N (%)	
Gender	
Male	14 (27.5)
Female	36 (70.6)
Other	1 (1.9)
Current Age	
Less than 35 years	15 (29.4)
35–39 years	29 (56.9)
40–44 years	7 (13.7)
Year of Fellowship Graduation	
2008–2010	3 (5.8)
2011–2014	14 (27.5)
2014–2016	34 (66.7)
Length of Fellowship Program	
3 years	38 (74.5)
4 years	13 (25.5)
Current Practice Setting	
Community based	5 (9.8)
University based	26 (56.9)
Community/Academic hybrid	17 (33.3)

Ninety-two percent of respondents felt comfortable writing a retrospective protocol and 74% felt comfortable drafting a letter of intent, but only 26% reported comfort writing a grant proposal. Even fewer (14%) felt comfortable writing an investigator initiated therapeutic trial. There were no differences in reported comfort levels by gender, age, time since fellowship, or length of fellowship.

**Fig. 1:**
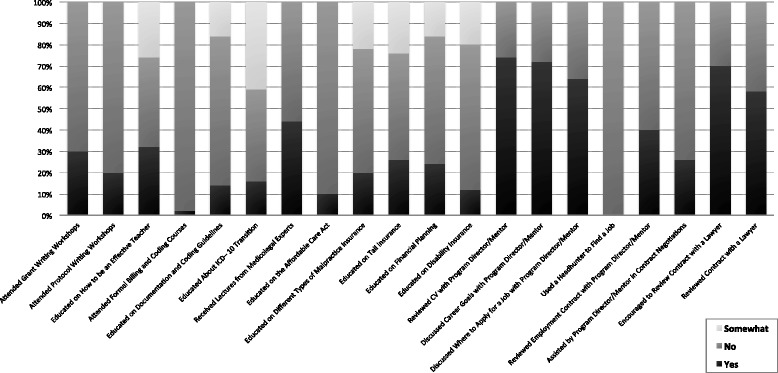
Candidate-reported exposure to educational and career development opportunities

Figure 1 shows candidate-reported exposure to educational opportunities and career development during their training. Twenty percent or fewer of respondents had attended a protocol writing workshop (20%) or billing and coding courses (2%), and reported little education in documentation and coding (14%), ICD-10 transition (14%), the Affordable Care Act (10%), malpractice insurance (20%), and disability insurance (12%).

**Fig. 2:**
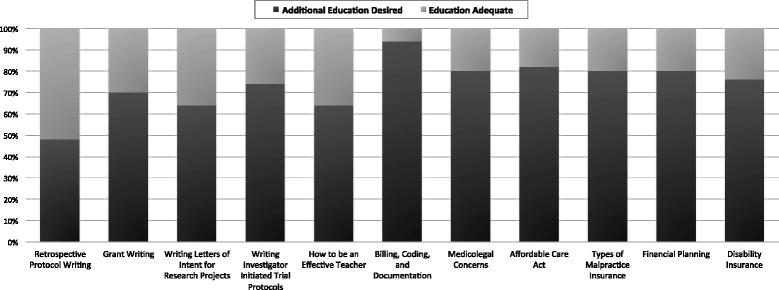
Candidate-reported desires for additional education by topic

Figure 2 shows areas in which candidate members reported whether or not they received adequate education on non-clinical topics during fellowship. The greatest proportions of candidate members wanted additional education in billing, coding, and documentation (94%) and the Affordable Care Act (82%). Topics about which additional education was least desired included retrospective protocol writing (48%), how to be an effective teacher (64%), and writing letters of intent (64%). Despite this, working in an academic practice was highly associated with the desire for more training in writing a letter of intent (OR 3.45 [95% CI 1.03–11.55], *p* = 0.04). Academic candidate members were also more likely to want additional training in drafting IIT, though this did not reach statistical significance (OR 2.97 [95% CI 0.80–10.90, *p* = 0.10). Age, length of fellowship, and time since completion of fellowship were not associated with reported interest in more education about any topic queried. Compared with males, a greater proportion of females wanted more teaching on billing and coding (79% vs. 100%, χ^2^ = 10.37, *p* = 0.005) and writing letters of intent (36% vs. 74%, χ^2^ = 6.40, *p* = 0.01). Males were less likely to want more training on disability insurance (OR 0.28 [95% CI 0.07–1.09], *p* = 0.059) and grant writing (OR 0.30 [95% CI 0.08–1.10], *p* = 0.07), though these did not reach statistical significance. Formal education in certain subject areas was negatively associated with a desire for further training on those subjects, including how to be an effective teacher (OR 0.11 [95% CI 0.03–0.45), *p* = 0.002), the Affordable Care Act (0.03 [95% CI 0.002–0.34], *p* = 0.004), and disability insurance (OR 0.22 [95% CI 0.06–0.87], *p* = 0.03). Of those who did not receive any teaching on the Affordable Care Act, 89% reported wanting more training.

Of the 48 fellowship program directors contacted, 19 completed the survey for a response rate of 40%. Sixty-five percent of the respondents directed three-year programs, while 35% directed four-year programs. The current didactic time for each program dedicated to the non-clinical practice of medicine is reported in Table 2. There were no associations between the length of the program and the amount of didactic time spent on any one topic. Program directors did indicate, however, that they would prefer more didactic time be spent on how to write an investigator-initiated trial compared to the current time allotted in their programs (χ^2^ = 6.11, *p* = 0.01).

**Table 2:** Program director report of current didactic time versus desired didactic time per topic (n = number of programs) over the entire length of fellowship

Educational Topic	Current Time 0–2 h (n)	Current Time ≥ 3 h (n)	Desired Time 0–2 h (n)	Desired Time ≥ 3 h (n)	χ^2^ p-value
Retrospective protocol writing	11	8	7	11	0.25
Writing letter of intent	12	7	7	11	0.14
Writing grant proposal	11	8	6	12	0.13
Writing investigator initiated trial	13	6	5	13	0.01
Being an effective teacher	10	9	5	13	0.12
Billing, coding, and documentation	12	7	7	11	0.14
Medical-legal concerns	12	7	9	9	0.42
Affordable Care Act	17	2	14	4	0.34
Types of malpractice insurance	17	2	13	5	0.18
Financial planning	17	2	13	5	0.18
Disability insurance	18	1	14	4	0.13

Table 3 shows candidate member and fellowship director responses to mentorship and job-hunting questions. Compared with fellowship directors, fewer candidate members reported that career goals were discussed (72% vs. 94%, χ^2^ = 3.89, *p* = 0.05) and that they received encouragement from their program directors to review employment agreements with a lawyer (70% vs. 94%, χ^2^ = 4.39, p = 0.05). Notably, those who were encouraged to review their contracts with a lawyer were more likely to actually do so compared with those who did not (OR 6.87 [95% CI 1.77–26.76], *p* = 0.005).

**Table 3:** Comparison of affirmative candidate member and program director experience of mentorship and job preparation

Item	Trainee	Program Director	Χ^2^ p-value
Faculty mentor or program director review CV	74%	72%	0.88
Faculty mentor or program director discuss career goals	72%	94%	0.05
Program director recommends where to apply for job	64%	78%	0.38
Faculty mentor or program director assist in contract negotiation	26%	50%	0.06
Fellows encouraged to review contracts with a lawyer	70%	94%	0.05

## Discussion

With increasing pressure on fellowship programs to train competent gynecologic oncologists in an era of rapidly advancing surgical and chemotherapeutic evolution, it is important to also recognize the concerns of trainees about adequate preparation for the practical aspects of being independent physicians. Our study shows that while recent fellowship graduates indicate a high level of comfort with some aspects of both the academic and business components of post-training employment, there remains a significant disparity in the perceived preparation for many of these important facets of practice.

The findings reported here are not unique to gynecologic oncology. In fact, other surgical specialties have reported a recognition of the importance of training in the business of medicine and a lack of trainee knowledge in this arena. Fakhry et al. [3] published the results of a survey of surgical residents and attending physicians, which included self-assessments of perceived knowledge and expertise in billing, coding, documentation, and insurance reimbursement. While 92% of residents reported that expertise in documentation and coding would make a difference in their practices, only 54% of residents accurately answered questions about billing correctly. Nearly 90% of both residents and faculty, however, thought further education on the topic was a crucial part of residency training. Similarly, a survey of general surgery program directors found that 87% agreed trainees should receive education in practice management [4]. Despite this, 34% of programs offered no training in the business of medicine, and 70% indicated that their residents were inadequately trained.

There are limited data on the importance of education in the more traditionally academic components of employment, such as preparation for protocol development and teaching. A survey of maternal fetal medicine fellows demonstrated that the percentage of respondents who felt they had adequate training to apply for grants was 20% [5], which is not dissimilar from the 26% reported here. However, assessments of other key components of an academic practice such as teaching and protocol development are lacking in the post-graduate medical literature. Multiple studies have highlighted the importance of mentorship, which likely serves as a surrogate for roles in university-based medicine. In both gynecologic oncology and maternal fetal medicine fellowship, having a close research mentor has been associated with completion of the thesis, fellowship satisfaction, and productivity [5, 6]. Having a faculty advisor or mentor has also been associated with a fellow’s desire to enter academic practice. In our study, the similarity in responses to questions between program directors and fellows regarding mentorship may suggest a close relationship between trainee and educator, though response bias cannot be ruled out as a factor in these findings. However, the discrepancy between the two groups regarding a discussion of career goals is striking. Faculty advisors and program directors may, therefore, need to be more explicit or transparent about their intentions for career counseling with trainees.

The amount of time dedicated to certain topics varies by program, and it is apparent that program directors have different opinions about the need for time spent to achieve competency. This is true of surgical training programs, as well, and is apparent in the diverse approach to education in the practice of non-clinical medicine. A number of strategies have been proposed to increase competency with contract negotiation, coding compliance, and financial planning. Jones, et al. [7] reported on a 10-lecture curriculum in managed care and coding compliance given over two years to surgical residents. Its efficacy was impressive; surgical coding compliance increased nearly 50% over a 12-month period. Other approaches have included weekend retreats and mock trial presentations, all of which demonstrated significant improvement in measured knowledge categories and high participant satisfaction [8, 9].

There are several weaknesses to this study. The survey that was released was developed de novo, and is not validated, so its reproducibility may be in question. The response rates of 28% and 40% for candidate members and fellowship directors, respectively, are low, but are in line with other studies requiring physician response to survey invitations. There is also a likely component of bias, as more than two-thirds of candidate respondents had less than three years of experience, and about 90% of had a university appointment or were in a community/academic hybrid practice. Relative overrepresentation of this population may skew perceptions on the need for additional academic-based professional training, but demonstrates that there are clearly concerns regarding tools for success as an academic gynecologic oncologist. Additionally, candidates were not queried about advanced training in research, business, or administration, which may have influenced their responses. Despite these limitations, this is the first study within the gynecologic oncology subspecialty to evaluate perceived preparation for measures outside of the usually studied surgical and medical oncology realm, and as such provides new insight into areas in which changes in didactic programs may be made. Larger investigations across multiple subspecialties, both on the residency and fellowship level, may assist in clarifying perceived additional educational need, with consideration being given to including such training as a core measure for trainee professionalism.

At our institution, we have initiated a more comprehensive program of didactics based on the current findings, now including lectures from risk management, billing and coding, and the institutional review board. Our approach, however, may not be universally applicable to all gynecologic oncology programs. In fact, since preparation in the business and academic practice of medicine is not a component of any core competency for training, creativity in curricula development should be encouraged. It is clear, though, that new graduates from gynecologic oncology fellowships feel inadequately prepared for some of the responsibilities they will have post-training. It remains incumbent on those entrusted to educate them to continuously strive to improve their programs and maintain open communication with the fellows so that a truly collaborative teaching environment can be developed.

## Conclusion

Recent gynecologic oncology fellowship graduates want more training in the non-clinical, academic and business practice of medicine. Modifying curricula to more effectively prepare graduates for professional lives should be considered. Improved dialogue between trainer and trainee will be a crucial component of such a curricular evolution.

## Additional files

Additional file 1: Candidate Member Survey. (DOCX 16 kb)

Additional file 2: Fellowship Program Director Survey. (DOCX 14 kb)

## Abbreviations

ABOG: American Board of Obstetrics and Gynecology
ACGME: Accreditation Council for Graduate Medical Education
LOI: Letters of Intent
SGO: Society of Gynecologic Oncology
