# Supplementary material for: Preparation in the business and practice of medicine: perspectives from recent gynecologic oncology graduates and program directors
Source: Gynecol Oncol Res Pract. 2017 Sep 22;4:14. doi: 10.1186/s40661-017-0051-z (PMC5610456; doi:10.1186/s40661-017-0051-z)
Supplement: Supplementary file 1 — Candidate Member Survey. (DOCX 16 kb) [file 40661_2017_51_MOESM1_ESM.docx]

Dear SGO Candidate Member:

We are conducting a nationwide questionnaire study of recent gynecologic oncology fellowship graduates to assess your educational experience in the business of medicine as a fellow. This study is being conducted solely at The University of Miami Sylvester Comprehensive Cancer Center, and has been approved by our Institutional Review Board, which has determined that the study meets ethical standards.

We are writing to invite you to take part in this study. Participation in this study involves completing a brief online questionnaire. The questionnaire will take about 15 minutes to finish, and your responses will be kept anonymous and reviewed only by the researchers involved in the study. Once you open the questionnaire, you will not be able to save your answers, so please plan on completing all questions in one sitting. *Please complete the questionnaire only* ***once****.*

Here is the link to the questionnaire:

[Survey Link]

If you are willing to participate in our study, please complete the online questionnaire *as soon as possible.* If you have any questions or concerns about the questionnaire or the study in general, please contact me at mschlumbrecht@miami.edu.

Thank you very much for considering this invitation to participate in our study. Your input will help us determine further ways to improve gynecologic fellowship training programs.

Sincerely,

Matthew Schlumbrecht, MD, MPH

Associate Director, Gynecologic Oncology Fellowship

Associate Professor, Division of Gynecologic Oncology

The University of Miami Sylvester Comprehensive Cancer Center

**Informed Consent Questionnaire Statement**

I have read the description of the study, and I have decided to participate in the research project described here. I understand that I may refuse to answer any (or all) of the questions at this or any other time. I understand that there is a possibility that I might be contacted in the future about this, but that I am free to refuse any further participation if I wish.

During the course of this study, the research team at The University of Miami Sylvester Comprehensive Cancer Center will be collecting information about me that they may share with health authorities, study monitors who check the accuracy of the information, and/or individuals who put all the study information together in report form. By answering the questions, I am providing authorization for the research team to use and share my information at any time. If I do not want to authorize the use and disclosure of my information, I may choose not to answer these questions. There is no expiration date for the use of this information as stated in this authorization.

**INSTRUCTIONS:**

Please read each question carefully and select the response that you feel most accurately represents your answer.

1. What is your gender?
   1. Male
   2. Female
   3. Other
2. What is your current age?
   1. Free text answer
3. What year did you graduate from your gynecologic oncology fellowship?
   1. Free text answer
4. What was the length of your gynecologic oncology fellowship program?
   1. 3 years
   2. 4 years
5. What is your current practice setting?
   - 1. Community based
     2. University based
     3. Community/Academic hybrid
6. Do you feel comfortable writing a retrospective protocol?
   1. Yes
   2. No
   3. Somewhat
7. Do you feel comfortable writing a letter of intent for a research project?
   1. Yes
   2. No
   3. Somewhat
8. Do you feel comfortable writing a grant proposal?
   1. Yes
   2. No
   3. Somewhat
9. Do you feel comfortable writing an investigator initiated therapeutic trial?
   1. Yes
   2. No
   3. Somewhat
10. Did you attend a grant-writing workshop as a fellow?
    1. Yes
    2. No
11. Did you attend any protocol writing workshop as a fellow?
    1. Yes
    2. No
12. Did you receive education on how to be an effective teacher to residents and students?
    1. Yes
    2. No
    3. Some
13. Did you go to a formal billing and coding course as a fellow?
    1. Yes
    2. No
14. Did you receive any education as part of your fellowship didactics in documentation and coding guidelines?
    1. Yes
    2. No
    3. Some
15. Were you provided education about the ICD-10 transition when it occurred?
    1. Yes
    2. No
    3. Some
    4. N/A
16. Did you ever receive a lecture from a lawyer/expert in medicolegal dealings?
    1. Yes
    2. No
17. Did you receive education about the Affordable Care Act?
    1. Yes
    2. No
18. Were different types of malpractice insurance explained to you?
    1. Yes
    2. No
    3. Some
19. Were you provided education about tail insurance?
    1. Yes
    2. No
    3. Some
20. Were you provided education, either formal or informal, about financial planning?
    1. Yes
    2. No
    3. Some
21. Were you provided education, either formal or informal, about disability insurance?
    1. Yes
    2. No
    3. Some
22. Did a faculty mentor or fellowship program director review your CV?
    1. Yes
    2. No
23. Did your fellowship program director of faculty mentor discuss with you specific career goals when deciding which jobs to apply to?
    1. Yes
    2. No
24. Did your fellowship program director make recommendations about where to apply for a job?
    1. Yes
    2. No
25. Did you use a headhunter to find a job?
    1. Yes
    2. No
26. After you received your contract, did you review your contract with a faculty mentor or your fellowship program director?
    1. Yes
    2. No
27. Did your fellowship program director or faculty mentor assist in contract negotiations with a prospective job?
    1. Yes
    2. No
28. Were you encouraged to review your contract with a lawyer?
    1. Yes
    2. No
29. Did you review your contract with a lawyer?
    1. Yes
    2. No
30. For each of the following, would you have liked to receive additional education (yes/no)?
    1. Retrospective protocol writing
    2. Grant writing
    3. Writing a letter of intent for a research project
    4. Writing an investigator initiated therapeutic trial protocol
    5. How to be an effective teacher
    6. Billing, coding, and documentation
    7. Medicolegal concerns
    8. Affordable Care Act
    9. Different types of malpractice insurance, including tail insurance
    10. Financial planning
    11. Disability insurance
