# Supplementary material for: Preparation in the business and practice of medicine: perspectives from recent gynecologic oncology graduates and program directors
Source: Gynecol Oncol Res Pract. 2017 Sep 22;4:14. doi: 10.1186/s40661-017-0051-z (PMC5610456; doi:10.1186/s40661-017-0051-z)
Supplement: Supplementary file 2 — Fellowship Program Director Survey. (DOCX 14 kb) [file 40661_2017_51_MOESM2_ESM.docx]

Dear Gynecologic Oncology Fellowship Program Director:

We are conducting a nationwide questionnaire study of Gynecologic Oncology fellowship program directors to assess the educational experience provided to your fellows in the business and practice of medicine. This study is being conducted solely at The University of Miami Sylvester Comprehensive Cancer Center, and has been approved by our Institutional Review Board, which has determined that the study meets ethical standards.

We are writing to invite you to take part in this study. Participation in this study involves completing a brief online questionnaire. The questionnaire will take about 15 minutes to finish, and your responses will be kept anonymous and reviewed only by the researchers involved in the study. Once you open the questionnaire, you will not be able to save your answers, so please plan on completing all questions in one sitting. *Please complete the questionnaire only* ***once****.*

Here is the link to the questionnaire:

[Survey Link]

If you are willing to participate in our study, please complete the online questionnaire *as soon as possible.* If you have any questions or concerns about the questionnaire or the study in general, please contact me at mschlumbrecht@miami.edu.

Thank you very much for considering this invitation to participate in our study. Your input will help us determine further ways to improve gynecologic fellowship training programs.

Sincerely,

Matthew Schlumbrecht, MD, MPH

Associate Director, Gynecologic Oncology Fellowship

Associate Professor, Division of Gynecologic Oncology

The University of Miami Sylvester Comprehensive Cancer Center

**Informed Consent Questionnaire Statement**

I have read the description of the study, and I have decided to participate in the research project described here. I understand that I may refuse to answer any (or all) of the questions at this or any other time. I understand that there is a possibility that I might be contacted in the future about this, but that I am free to refuse any further participation if I wish.

During the course of this study, the research team at The University of Miami Sylvester Comprehensive Cancer Center will be collecting information about me that they may share with health authorities, study monitors who check the accuracy of the information, and/or individuals who put all the study information together in report form. By answering the questions, I am providing authorization for the research team to use and share my information at any time. If I do not want to authorize the use and disclosure of my information, I may choose not to answer these questions. There is no expiration date for the use of this information as stated in this authorization.

**INSTRUCTIONS:**

Please read each question carefully and select the response that you feel most accurately represents your answer.

1. What is the length of your gynecologic oncology fellowship program?
   1. 3 years
   2. 4 years
2. By the time they graduate, how much time is provided in a structured format to educate your fellows on the following: (0 hours, 1-2 hours, 3-4 hours, more than 4 hours)
   1. Retrospective protocol writing
   2. Writing a letter of intent for a research project
   3. Drafting a grant proposal
   4. Writing an investigator initiated therapeutic trial protocol
   5. How to be an effective teacher to residents and students
   6. Billing, coding, and documentation
   7. Medicolegal concerns
   8. Affordable Care Act
   9. Different types of malpractice insurance, including tail insurance
   10. Financial planning
   11. Disability insurance
3. Ideally, how much time to you think should be dedicated to providing education on each of the following topics? (0 hours, 1-2 hours, 3-4 hours, more than 4 hours)
   1. Retrospective protocol writing
   2. Writing a letter of intent for a research project
   3. Drafting a grant proposal
   4. Writing an investigator initiated therapeutic trial protocol
   5. How to be an effective teacher to residents and students
   6. Billing, coding, and documentation
   7. Medicolegal concerns
   8. Affordable Care Act
   9. Different types of malpractice insurance, including tail insurance
   10. Financial planning
   11. Disability insurance
4. Do faculty mentors or fellowship program director review fellow CVs?
   1. Yes
   2. No
5. Does the fellowship program director of faculty mentor discuss specific career goals when deciding which jobs a fellow should to apply to?
   1. Yes
   2. No
6. Does the fellowship program director make recommendations about where to apply for a job?
   1. Yes
   2. No
7. Does the fellowship program director or faculty mentor assist in contract negotiations with a prospective employer?
   1. Yes
   2. No
8. Are fellows encouraged to review employment agreements/contracts with a lawyer?
   1. Yes
   2. No
